# Recent progress of nano-drugs in neutron capture therapy

**DOI:** 10.7150/thno.95034

**Published:** 2024-05-19

**Authors:** Yi-Tong Zhou, Kai Cheng, Bo Liu, Yuan-Cheng Cao, Jin-Xuan Fan, Zhi-Gang Liu, Yuan-Di Zhao

**Affiliations:** 1 Britton Chance Center for Biomedical Photonics at Wuhan National Laboratory for Optoelectronics - Hubei Bioinformatics & Molecular Imaging Key Laboratory, Department of Biomedical Engineering, College of Life Science and Technology, Huazhong University of Science and Technology, Wuhan 430074, Hubei, P. R. China; 2 NMPA Research Base of Regulatory Science for Medical Devices & Institute of Regulatory Science for Medical Devices, Huazhong University of Science and Technology, Wuhan 430074, Hubei, P. R. China; 3 Cancer Center, the 10th Affiliated Hospital of Southern Medical University (Dongguan People's Hospital), Southern Medical University, Guangzhou 510280, China; 4 Dongguan Key Laboratory of Precision Diagnosis and Treatment for Tumors, Dongguan Engineering Research Center for Innovative Boron Drugs and Novel Radioimmune Drugs, the 10th Affiliated Hospital of Southern Medical University, Guangzhou 510280, China; 5 State Key Laboratory of Advanced Electromagnetic Engineering and Technology, Huazhong University of Science and Technology, Wuhan, Hubei 430074, P. R. China

**Keywords:** neutron capture therapy, nano-drugs, multi-functional compound boron drug, gadolinium-based drugs, anti-tumor therapy

## Abstract

As a developing radiation treatment for tumors, neutron capture therapy (NCT) has less side effects and a higher efficacy than conventional radiation therapy. Drugs with specific isotopes are indispensable counterparts of NCT, as they are the indespensable part of the neutron capture reaction. Since the creation of the first and second generations of boron-containing reagents, NCT has significantly advanced. Notwithstanding, the extant NCT medications, predominantly comprised of small molecule boron medicines, have encountered challenges such monofunctionality, inadequate targeting of tumors, and hypermetabolism. There is an urgent need to promote the research and development of new types of NCT drugs. Bio-nanomaterials can be introduced into the realm of NCT, and nanotechnology can give conventional medications richer functionality and significant adaptability. This can complement the advantages of each other and is expected to develop more new drugs with less toxicity, low side effects, better tumor targeting, and high biocompatibility. In this review, we summarized the research progress of nano-drugs in NCT based on the different types and sources of isotopes used, and introduced the attempts and efforts made by relevant researchers in combining nanomaterials with NCT, hoping to provide pivotal references for promoting the development of the field of tumor radiotherapy.

## 1. Introduction

Conventional radiation is seen as one of the best methods for treating cancer 1. The double-strand DNA of cancer cells is directly damaged by high-frequency radiation (such as gamma rays and X-rays) at the tumor location, which prevents the cell from continuing its reproduction and ultimately results in cell death 2. Nevertheless, the high-energy radiation unavoidably damages the surrounding tissue and organs in addition to eliminating cancer cells. Thankfully, this issue can be resolved by relocating the radiation generating site within the cancer cells 3. Unlike conventional radiation, neutron capture therapy (NCT) produces high-energy radiation from inside tumors by inducing a neutron capture reaction within the tumor cells 4. This method produces secondary radiation and recoil particles that have a range equivalent to the size of the cells (5-9 µm) and an exceptionally high linear energy transfer (LET), which is the rate of energy loss along an ionizing particle's path. As a result, they will hardly affect nearby normal tissues 5-7.

The occurrence of neutron capture reaction requires two essentials: neutron source and nuclides with large neutron capture cross sections 8. According to different energies, neutrons can be divided into three types, thermal neutrons, epithermal neutrons and fast neutrons. Among them, fast neutrons (En ≥ 10 keV) have the highest energy, but their efficiency in nuclear reactions is relatively low, so thermal neutrons (En ≤ 0.5 eV) and epithermal neutrons (0.5 eV < En < 10 keV) are selected as the neutron source of NCT. Among the natural abundant stable and non-radioactive isotopes, ^10^B, ^155^Gd and ^157^Gd have the largest thermal neutron capture cross section (Table 1), making them appropriate for developing NCT medicine 9. Boron neutron capture therapy (BNCT) is based on the reaction between ^10^B and thermal neutrons, generating alpha particles and ^7^Li with high LET of 190 keV/µm and 160 keV/µm, which means energy of several alpha particles could nearly destroy any cell of human body. The equations for the neutron capture reaction of ^10^B and ^157^Gd are as follows.


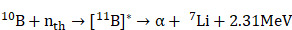

(1)


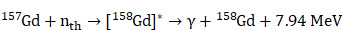

(2)

Compared to BNCT, the principle of gadolinium neutron capture therapy (GdNCT) is slightly different. The reaction produces uncharged gamma rays along with internal conversion and Auger electrons, which can damage DNA within a cell diameter 10. In comparison with^ 10^B, ^157^Gd has larger neutron capture cross section and a wider variety of secondary particles after reacting with neutrons. Meanwhile, gadolinium is a paramagnetic which is suitable for using as magnetic resonance imaging (MRI) contrast agent. Using ^157^Gd as NCT medicine can combine MRI with GdNCT, achieving more precise and personalized treatment.

Since the development of BNCT requires both neutron generating devices and boron-containing drugs, it has not been greatly improved before the vigorous development of modern pharmaceutical technology. BNCT has now developed to the stage of clinical treatment, many countries such as the United States 11, Germany 12, Italy 13 and so on, have carried out BNCT as a clinical choice in the past few years. According to the statistics published by researchers from Japan, the median overall survival after BNCT of brain tumor post-BNCT and post-diagnosis were 29.6 months and 98.4 months 14. According to statistics, BNCT is mainly used in the clinical treatment of head and neck tumors so far. In addition, it has also been tried in melanoma, lung cancer and hepatocellular carcinoma 15. The development of boron containing medicine used in BNCT has gone through three generations 16. The first generation BNCT drugs are represented by borax, boric acid and their derivatives, although they could accomplish NCT to some extent, they have severe toxicity, several side effects and lack of tumor-specificity 17. To address this, the second-generation radionuclide drugs represented by 4-dihydroxyboryl-L-phenylalanine (BPA) and sodium borocaptate (BSH) soon came into being 18. Compared with the first-generation boron drugs, BPA and BSH can be preferentially taken up by tumor cells and can effectively increase the boron concentration in tumor cells 19. In order to reach the idealized therapeutic effect of NCT, drugs containing ^10^B and ^157^Gd have to meet several needs as below. Firstly, the NCT drugs should be able to target tumor cells accurately, minimizing retention in normal tissues and cells 20. Secondly, the basic value of ^10^B concentration should achieve 20-30 µg/g tumor tissue and tumor-to-blood (T/B) boron ratio of 3.0 at least would exert BNCT effectively, and 50-200 µg ^157^Gd /g tumor tissue for GdNCT to kill tumor cells. Finally, the ideal ^10^B and ^157^Gd carrier agent also needs to achieve quantitative release within tumor tissue and traceable imaging, be able to be mass-produced, possess great chemical stability, and be easy to store for a long-time.

In order to address these issues, third-generation BNCT drug research is under underway, with a primary focus on BNCT nanodrugs and biomolecules containing boron 21. Nanomedicine involves preparing active chemicals, such as pharmaceutical components, into nanoscale particles or combining chemotherapeutic medications with suitable carrier materials to create pharmacological preparations on a nanoscale. At the scale of 10-1000 nm, the physical and chemical properties, pharmacodynamics (PD), and pharmacokinetics (PK) show obvious differences from conventional preparations. For example, nanoscale particles can enter the interior of cells via transcytosis rather than free diffusion; Surface modification of nanoparticles can significantly improve its biocompatibility and systemic circulation time; Specific tumor-targeting molecule coupling on the surface of nanostructures can achieve tumor-targeted delivery and controlled release of drugs and so on 22, 23. Considering the rich functions and strong plasticity of nanomedicines, the introduction of biological nanomaterials in the field of NCT can overcome plenty of shortcomings. Furthermore, researchers combine nanotechnology with compounds such as peptide, antibody, polymer conjugation, polymer micelles, which brings new hope to achieve higher efficacy and wider application range of NCT 24, 25. Different isotopes from various sources have unique properties and uses. Different isotope types, like ^10^B and ^157^Gd, can be successfully hybridized and mixed to create complementary advantages by building nanomedicines. Currently, the design of nano-sized drugs for NCT can start from the following aspects: 1) Loading ^10^B or ^157^Gd containing compounds into nanocarriers for drug delivery; 2) Preparation of nano-scale NCT drugs and multifunctional modification to give them tumor targeting, high biocompatibility and other functions; 3) Incorporation of certain isotopes into other nano-contrast agents to achieve integrated diagnosis and treatment with simultaneous treatment and imaging 26-28.

In this review, we list and categorize the ^10^B and ^157^Gd-containing medications that have been used in NCT to date, evaluating their inventiveness and design flaws. And we continue to discuss the use of nanomedicine in the field of NCT and present potential obstacles for future progress based on these efforts and experiences.

## 2. Boron-containing NCT Nanomedicine

Boron (B) is a non-metallic element that mainly exists in borax ore in nature abundantly. Natural boron (content of ^10^B is about 19.9%) has a reaction cross section of 752 barn, which can be directly used as a neutron absorber, while ^10^B has a higher thermal neutron absorption cross section of 3837 barn. After the radiation of thermal or epithermal neutron beams, ^10^B can undergo a neutron capture reaction, releasing helium and lithium atoms with high energy up to 2.31 MeV, thereby killing tumor cells. ^10^B is one of the non-radioactive isotopes of B, which has strong affinity with tumor cells. Furthermore, the products after neutron capture reaction are also non-radioactive and stable. Therefore, compounds containing ^10^B are now being widely used in NCT. The idea of BNCT was first proposed by American biophysicist Locher as early as 1936 based on the principle of ^10^B capturing thermal neutrons. However, due to the limitations of both the boron carriers and thermal neutron generators, the development of BNCT is relatively slow. During the revolution of boron-containing drugs for nearly a century, tumor specificity and targeting have always been the goal of researchers' design and development. The second-generation boron drugs represented by BPA and BSH are currently the preferred boron carriers in clinical applications 29. In recent years, with the continuous improvement of drug synthesis technology, clinical diagnosis and tumor treatment, the shortcomings of the second-generation boron drugs in tumor targeting and specificity are expected to be further improved, which also led to the vigorous development of the third-generation boron carrier with nanomedicine as one of the core members 30.

### 2.1 Organic Boron Sources

#### 2.1.1 BPA&BSH

The structural characteristics of BPA is similar to tyrosine, a conditionally essential amino acid for human body. Inspired by the metabolic pathway of amino acids in human body, BPA is involved in the synthesis of related proteins on tumor cell membranes, which makes it able to selectively accumulate in tumor cells' during blood circulation, and finally realizes the specific combination of BPA and tumor site 31. In addition, LAT1 is a high-affinity amino acid transporter, which is overexpressed in blood-brain barrier; thus, BPA has the potential to target tumors in the brain such as gliomas after crossing the blood-brain barrier (BBB) 32.

**Figure 2:**
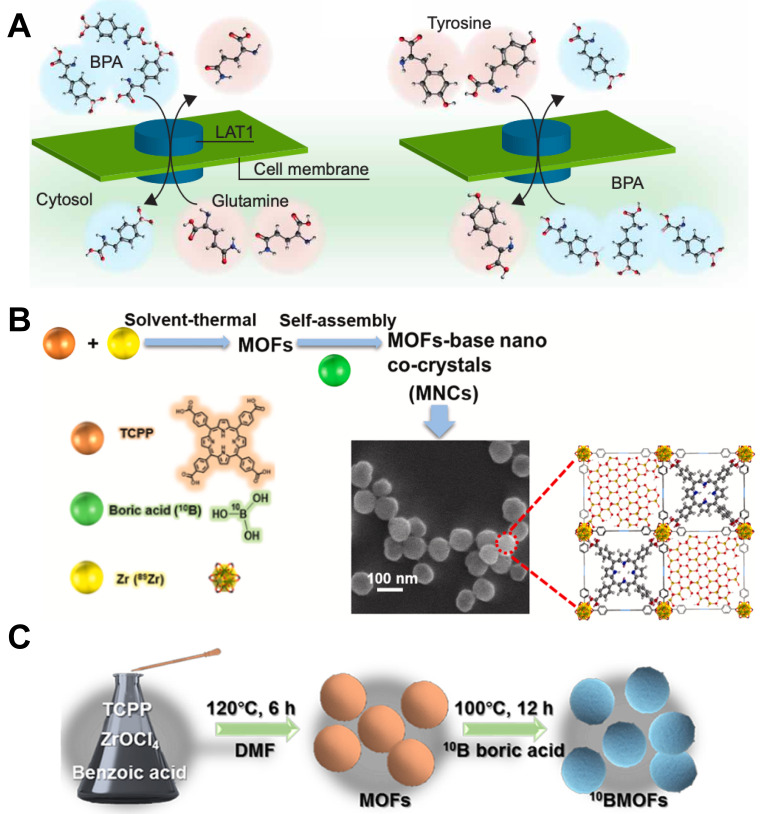
Designation of BPA&BSH containing BNCT nano-drugs. (A) Cell internalization of BPA through the LAT1 transporter and efflux of intracellular BPA. Reproduced with permission from 33. Copyright 2020, American Association for the Advancement of Science. (B) MOF-base nano co-crystals' design strategy. Reproduced with permission from 35. Copyright 2022, Elsevier. (C) Schematic representation of the synthesis of BMOFs. Reproduced with permission from 36. Copyright 2024, American Chemical Society.

BPA is already the most effective boron carrier in clinical treatment. BPA-based nanoparticles in terms of polymers or micelles can offer boron atoms larger density and add more functions through surface modification and functionalization. Directly using BPA to synthesize new nanoparticles can improve its shortcomings on the basis of its advantages such as fast metabolism, high toxicity, and lack of targeting. However, due to the antiport mechanism, BPA is easy to efflux from the cytoplasm, making it hard to maintain a therapeutic boron concentration in the tumor site. To solve this problem, Nomoto *et al.* formed a complex using poly (vinyl alcohol) (PVA) and BPA, which did not change the inherent structure of BPA, so it could still be endocytosed into tumor cells through the LAT1 (Figure 2A). In addition, PVA can increase the solubility of BPA in aqueous solution, thereby prolonging the retention of PVA-BPA in tumors and facilitating the rapid clearance of the nanomedicine from the bloodstream and normal organs, so that PVA-BPA exhibited obvious antitumor activity in *in vivo* experiments of CT26 tumor models 33, 34.

**Figure 3:**
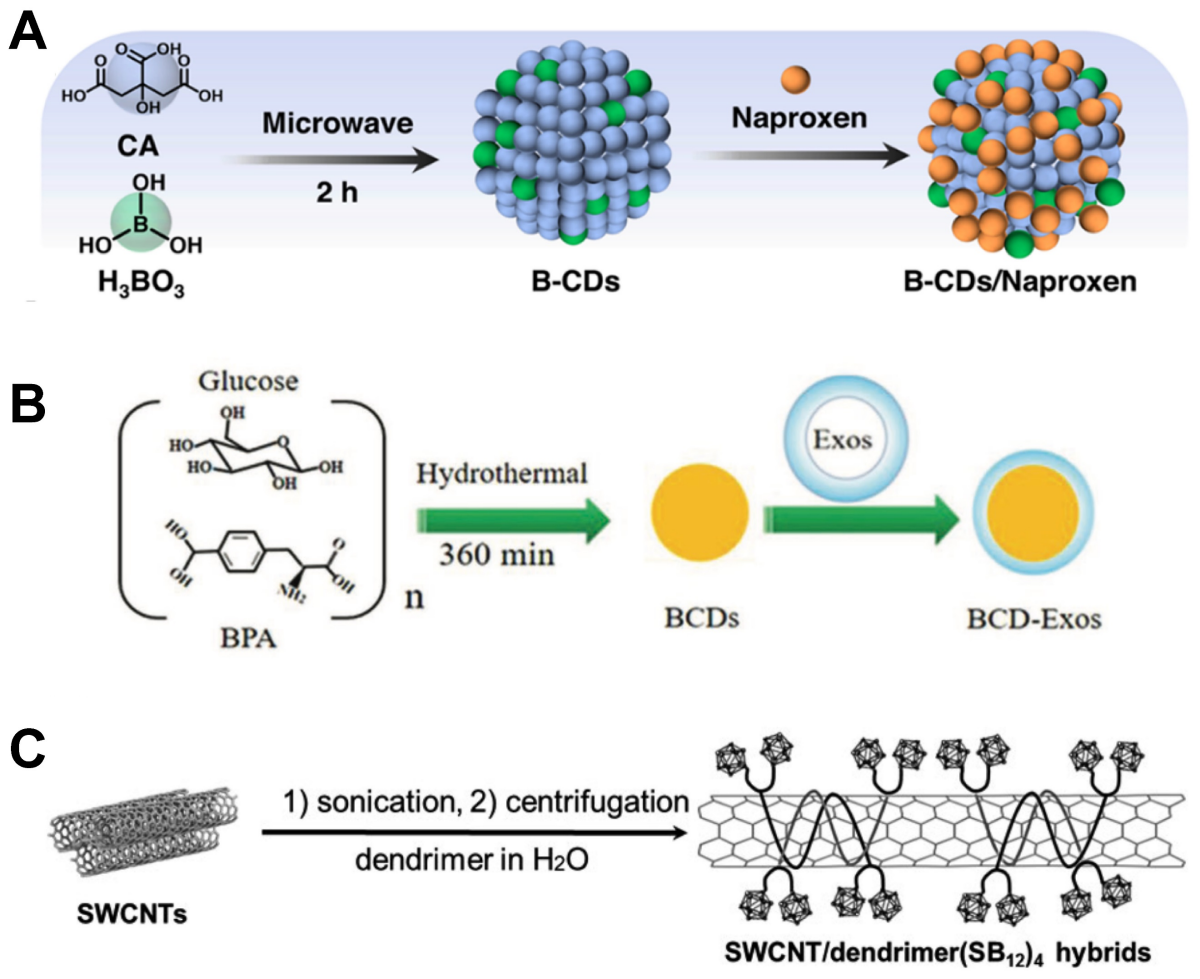
Designation of BPA&BSH containing BNCT nano-drugs. (A) Synthesis preparation with B-CDs/Naproxen. Reproduced with permission from 38. Copyright 2023, American Chemical Society. (B) The preparation of BCD-Exos. Reproduced with permission from 40. Copyright 2021, Wiley-VCH GmbH. (C) Fabrication of the SWCNT/dendrimer (SB_12_)_4_ nanohybrids. Reproduced with permission from 41. Copyright 2021, Wiley-VCH GmbH.

In addition to common tumors such as colon cancer and breast cancer, BNCT is considered to be one of the most feasible ways to treat brain glioma (Figure 2B,C) 35, 36. Because of the unique anatomical structure and physiological characteristics of the brain, traditional antineoplastic drugs have difficulty reaching the brain glioma, and the effect of traditional treatment methods is limited. If a boron carrier that can efficiently pass through BBB and target brain glioma can be designed, and highly accumulate in tumor cells to reach the concentration of boron required for treatment, it is expected to use BNCT to overcome the treatment conundrum of brain glioma 37. To this end, Li *et al.* synthesized a new type of carbon dots (CDs) that contains boron using _D_-glucose and BPA as raw materials. CDs are nanoparticles that have excellent biocompatibility and optical properties which can be visualized in real time with fluorescence imaging (Figure 3A) 38. To cross BBB and prolong the retention in tumor sites, exosomes from circulating macrophages were coated outside the boron-containing CDs to keep its structural integrity while crossing the BBB 39. Experiments using U87MG glioma cells showed that such novel CDs distributed around the nuclei of the tumor cells after intravenous administration. By adjusting the biodistribution of boron, this nanoparticle enhanced T/N ratio and realized the precise treatment of BNCT with invasive fluorescence imaging (Figure 3B) 40.

Similar to BPA, BSH is also one of the typical boron carriers of the second-generation. Differently, BSH was developed from the carborane chemistry, which has more than 10 boron atoms in one single molecule, and results in significant lower toxicity based on its boron content. Single-walled carbon nanotubes (SWCNTs) are nanomaterials that can be used in *in vivo* fluorescence optical imaging and can be used as carriers for drug delivery due to their structural specificity. Yamagami *et al.* grafted BSH onto poly(amido)amine (PAMAM) dendrimer, and then used SWCNTs as a nano-carrier to attach the dendrimer onto it, resulted in a SWCNTs/B_12_-cluster nanohybrid (Figure 3C). The advantage of using SWCNTs as a drug carrier is that it can emit fluorescence of NIR-Ⅱ, which in turn can achieve noninvasive fluorescence imaging *in vivo* while drug delivering, then realize the integration of imaging and therapy 41.

#### 2.1.2 Other Organic Boron Sources

Compared to inorganic boron sources, organic boron-containing compounds are relatively fewer. Several cases of boron-containing nanomedicines derive from organic boron sources that are applied in BNCT basically following the rule of incorporating inorganic boron compounds into organic molecules like liposomes, proteins and so on. Liposomes are artificial membranes with a phospholipid bilayer structure. In water, the hydrophilic head of the phospholipid molecule is inserted into the water, and the hydrophobic tail extends into the air. It can be used for genetic modification and the preparation of various kinds of drugs, and can deliver drugs into the cell by using its ability to fuse with cell membrane. Kueffer *et al.* used liposomal delivery and designed a boron-rich liposome which could combine the merits of both outstanding drug delivery capacity and high boron content. Researchers chose two boron agents Na_3_[1-(2′-B_10_H_9_)-2-NH_3_B_10_H_8_] (TAC) and K[*nido*-7-CH_3_(CH_2_)_15_-7,8-C_2_B_9_H_11_] (MAC). The former was a hydrophilic polyhedral borane which was encapsulated in the aqueous core of the liposome, while the latter was a lipophilic boron agent that was added into the bilayer membrane structure. The borane-containing liposome was tested on the model of localized EMT6 solid flank tumors in BALB/c mice, and it showed effective antitumor response with no obvious systematic toxicity or side effects, suggesting that it could be a promising boron agent in BNCT 42. Moreover, Maitz *et al.* used the same material to verify its therapeutic effect on another cancer model and made a comparison at the same time. Results showed that the boron-rich liposome could reach a considerable boron concentration in the CT26 tumors and further achieve BNCT efficacy as same as in the EMT6 tumors 43.

As early as 1994, there were researchers who designed boron-contaning dendrimers to perform BNCT. Barth *et al.* took advantage of different properties of dendrimers from other oligomer macromolecules, such as functional density, size and branching, to use them as macromolecular carriers for ^10^B. In addition, they took use of boronated monoclonal antibodies (MoAbs) and ^125^I to locate the boronated MoAbs *in vivo* 44. Using dendrimers as boron carriers could take advantage of their nano-size, which saves the trouble of designing other borax nanoparticles, and this work also provides ideas for the design of the subsequent boron nanoparticles in the following research. In a similar way, Barth *et al.* synthesized boronated dendrimers linked to epidermal growth factor (EGF) in order to implement BNCT targeting gliomas 45. Such strategy can be extended to various types of tumors, by changing the proteins or antibodies linked to the dendrimers.

**Figure 4:**
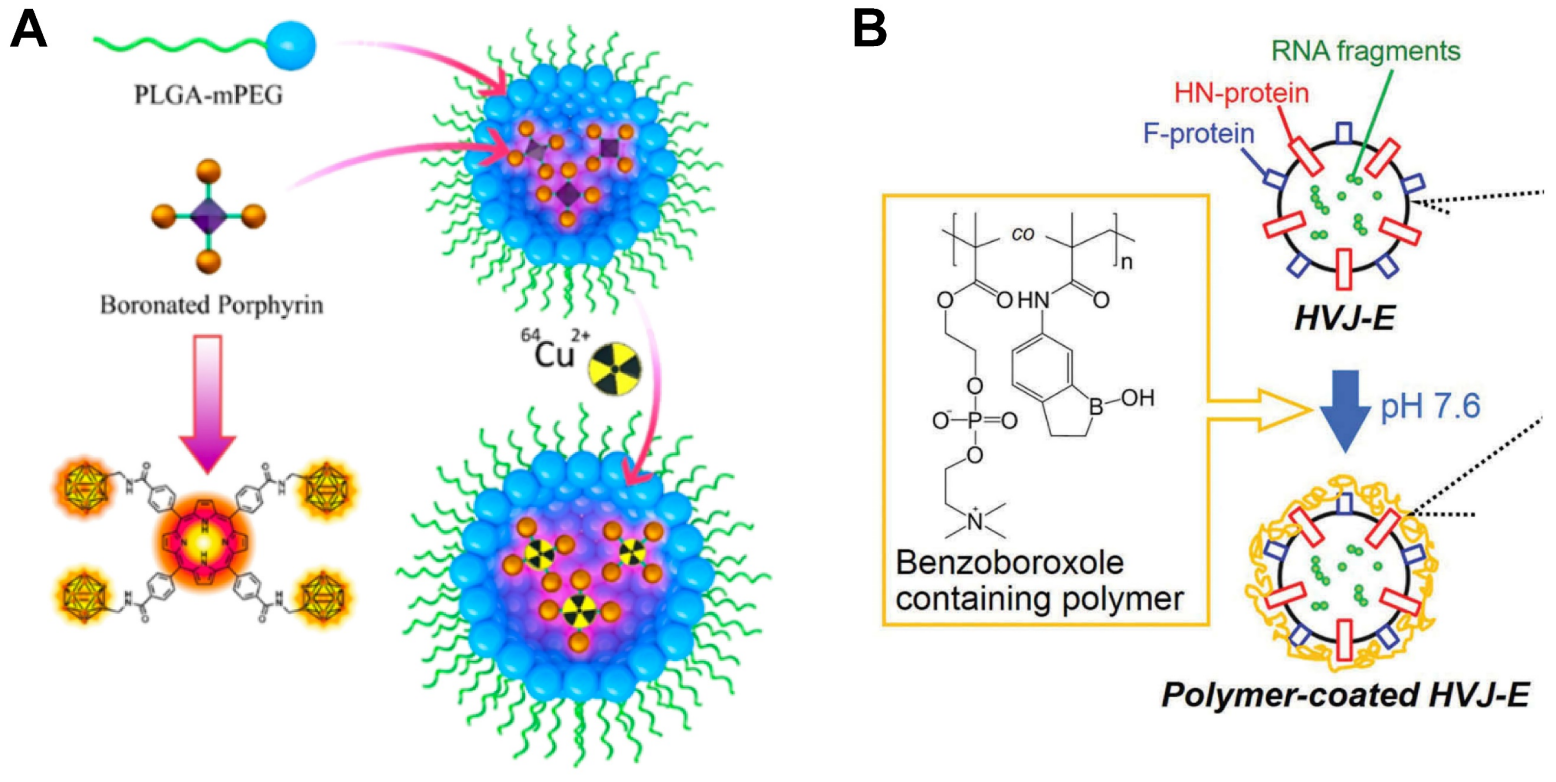
Designation of ^10^B containing BNCT nano-drugs with organic boron source. (A) Schematic illustration of imaging-guided BNCT with BPN. Reproduced with permission from 46. Copyright 2018, American Chemical Society. (B) Schematic illustration of HVJ-E surface modification by benzoxaborole-containing polymer. Reproduced with permission from 47. Copyright 2019, Taylor & Francis.

Porphyrin is one of the macromolecule heterocyclic compounds that possesses certain photosensitive properties. Porphyrins can form various complexes with many kinds of metal ions in nature, also, various types of porphyrins with different functions are available through modern synthesis and designation. Shi *et al.* presented a boronated porphyrin nanocomplex with particularly promising properties which could also be labelled with Cu-64 in order to achieve imaging guided BCNT (Figure 4A). Interestingly, they figured out that compared to one single injection with relatively high dose of boron agent, multiple injections may result in better tumor uptake and lower toxicity. Taking melanoma tumor as an example, the boronated porphyrin exhibited almost complete tumor suppression and showed great potential for delivering boron in BNCT 46.

Some viruses and bacteria can also act as drug carriers after being inactivated. Hemagglutinating virus of Japan (HVJ) is a virus that was once mainly used in cell fusion technology in cell engineering. The basic principle is that it contains binding sites for cell surface receptors, which can promote the aggregation of different cells and ultimately allow the cell membranes to fuse with each other. There are mainly two types of proteins that promote the fusion of membranes: fusion (F) and hemagglutinin-neuraminidase (HN) proteins. However, HN proteins can easily fuse with erythrocyte and further cause hemagglutination and hemolysis which may be harmful to the normal tissues and organs. To solve this severe problem, Yoneoka *et al.* tried to coat the HVJ-E with a copolymer layer which contains boron, so that they could combine BNCT with immunotherapy at the same time (Figure 4B). The inactivated HVJ still possessed immunogenicity, and the therapeutic effect appeared to be more powerful and could even suppress the occur of metastatic tumors with the participation of BNCT 47.

### 2.2 Inorganic Boron Sources

#### 2.2.1 Carborane and its Derivatives

**Figure 5:**
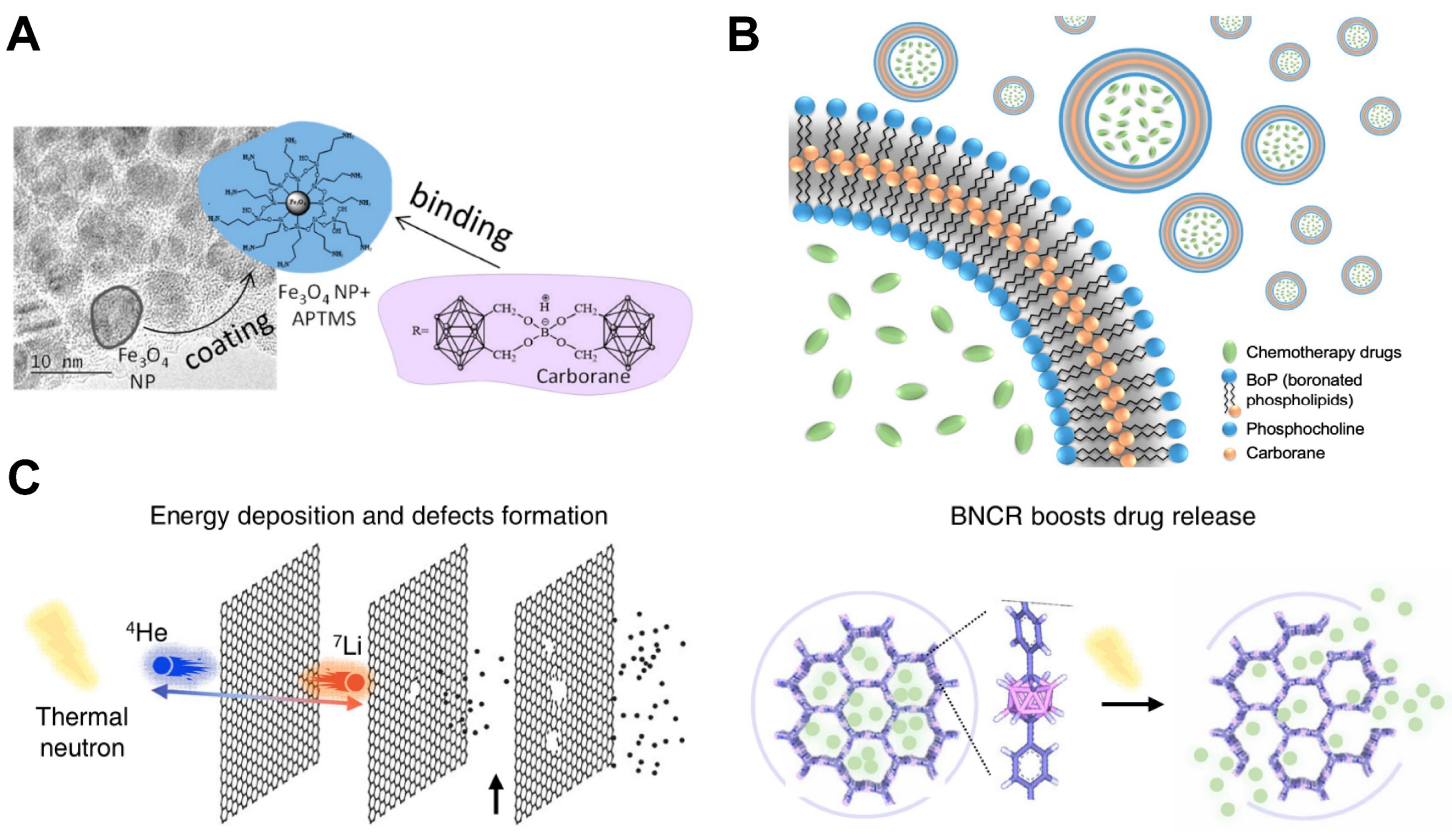
Designation of ^10^B containing BNCT nano-drugs with carborane and its derivatives. (A) Schematic representation of the synthesis of the Fe_3_O_4_ nanoparticle binding APTMS. Reproduced with permission from 51. Copyright 2019, MDPI (Basel, Switzerland). (B) Schematic illustration of chemotherapeutic agents encapsulated carboranyl-lipid boronsomes. Reproduced with permission from 56. Copyright 2022, Springer Nature. (C) The simulation of energy deposition and defects formation of BNCT and the schematic representation of drug release. Reproduced with permission from 57. Copyright 2023, Springer Nature.

Carborane is a product in which some boron atoms in polyhedral borane molecules are replaced by carbon atoms. Although it contains C-H bonds, it belongs to the category of inorganic compounds. According to the number of electrons in the skeleton and different molecular structure, it can be classified into a few different types, such as closo-carborane, nido-carborane, arachno-carborane, etc., which have unique chemical properties and are quite different from traditional hydrocarbons. Carborane and its derivatives have high boron atom content, which contain 10-21 boron atoms in a single molecule, after further functionalization, carborane can be regarded as one of the most ideal choices of boron carriers in BNCT 48, 49. The ultra-small size and magnetic properties of magnetic nanomaterials represented by metal nanoparticles and metal nanotubes make them suitable for intravenous administration. The main limitation of BNCT now is that boron-containing compounds need to selectively accumulate in the tumor site and reach a significant concentration. Korolkov *et al.* applied magnetic-nanomaterials to solve this bottle-neck problem. They synthesized a nanotube containing Ni and Fe as a carrier, modified carborane derivatives on it through covalent and ionic reactions, and coated it with (3-Aminopropyl)trimethoxysilane (APTMS) to improve its biocompatibility and reduce toxicity, finally realized magnetic field-induced targeted drug delivery 50. Using the same strategy, this research team also attached carborane compounds to Fe_3_O_4_ magnetic nanoparticles, with APTMS coating on the outside to improve its characteristics (Figure 5A). A stable nanoplatform for BCNT drug delivery was constructed by utilizing the advantages of Fe_3_O_4_ super-paramagnetism and high surface area 51.

Boron-containing compounds can be combined with various polymers or copolymers to improve their properties and adapt to different application scenarios 52-54. Block copolymers can self-assemble into polymer micelles in water, they are simple to prepare, have high biocompatibility, good stability and strong water solubility. Its hydrophobic core can load poorly soluble drugs for solubilization and has strong drug loading capacity, which can be widely used as a practical nanocarrier for drug imaging and targeted delivery. Zhang *et al.* developed a polymeric micelle containing carborane clusters with high selectivity for hepatoma cell HepG2 cells and low toxicity. This micelle could self-assemble by PEGylated galactose polymer and could target Asialoglycoprotein receptor (ASGP-R), which is one of the most ideal targets for hepatocyte-specific delivery. The results of *in vitro* experiments showed that after thermal neutron beam irradiation, the cytoskeleton of HepG2 cells changed significantly, the ability to migrate was weakened, and the double strands of DNA were destroyed. It could be seen that when designing new nanomedicine, the disease model to be matched can be fully considered, which could provide a reference for the synthesis paths of drugs 55.

Nano-boron delivery agents designed for BNCT can also be realized by modifying other biomass with boron compounds, such as adding boron to liposomes, vesicles and other substances, in order to achieve the advantages of both. The combination of these materials can solve the limitations of existing boron carriers in terms of biocompatibility and *in vivo* stability. Li *et al.* synthesized a boronsome, a carboranyl-phosphatidylcholine based liposome, to realize the combination therapy of chemotherapy and BNCT (Figure 5B). Different from the previous strategy of using liposomes to load drugs, they used boron-containing moiety to modify the hydrophilic head of phospholipids, so that the liposomes themselves contained boron elements, and the internal cavity was emptied to load drugs that related to other means of therapy, both improving the boron content of nanoparticles and can be further combined with other imaging and therapeutic methods. In addition, the researchers used ^64^Cu to label the boronsome, so they were able to use PET imaging to track the nanoparticle* in vivo*, and verified its ability to specifically accumulate and retain in tumor sites. Furthermore, the therapeutical effect was further amplified after encapsulating poly ADP-ribose polymerase (PARP) inhibitors, which can interfere the reparation of DNA double strand 56. Similar to this strategy, Shi *et al.* also put boron into the drug carrier to prepare a neutron-activated boron capsule which made up of carborane-based covalent organic framework loaded with immune adjuvant inside (Figure 5C). After being irradiated with thermal neutron beams, since the drug-loaded framework contains boron, the carrier would undergo a capture reaction to accelerate the release of the immune adjuvants which could lead to effective anti-tumor immune response. Previous studies have proven that radiotherapy has connections with mechanisms related to the immune system, and combinational therapy may promote the abscopal effect that suppress metastasis. Based on the above findings, the researchers combined BNCT with immunotherapy. On the basis of BNCT activating the body's immune response, the immune adjuvants were further released to prevent the self-repair of cancer cells, to improve the therapeutic effect, and to make up for the limitations of the single treatment mode 57.

#### 2.2.2 Boron Nitride

**Figure 6:**
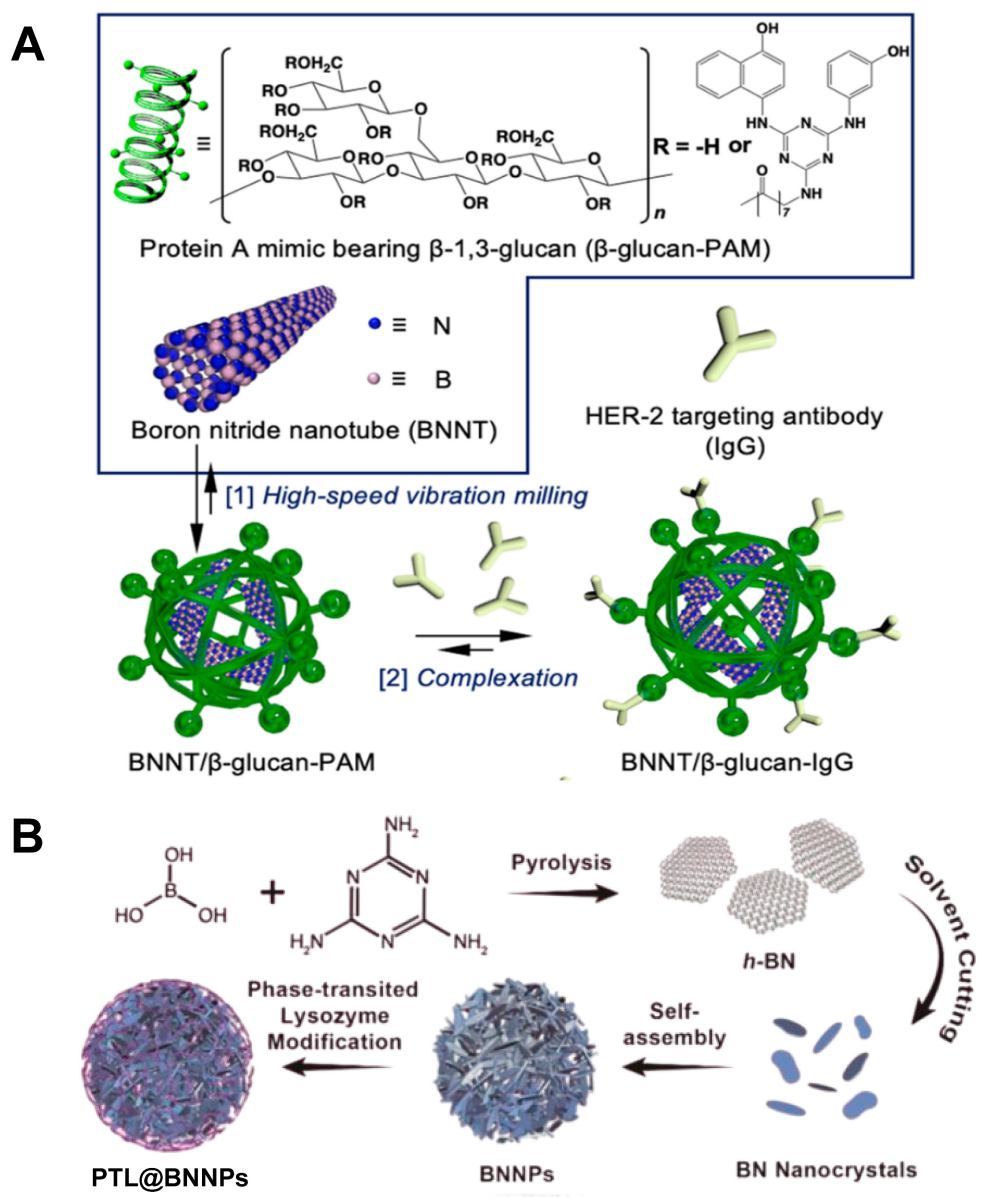
Designation of ^10^B containing BNCT nano-drugs with boron nitride. (A) Schematic illustration of the two-step method used to prepare the tumor-selective BNNT/b-glucan complex conjugated to a HER-2 targeting antibody (BNNT/b-glucan-IgG). Reproduced with permission from 60. Copyright 2023, Royal Society of Chemistry. (B) Schematic representation of the synthesis of the PTL@BNNPs. Reproduced with permission from 62. Copyright 2019, American Chemical Society.

**Figure 7:**
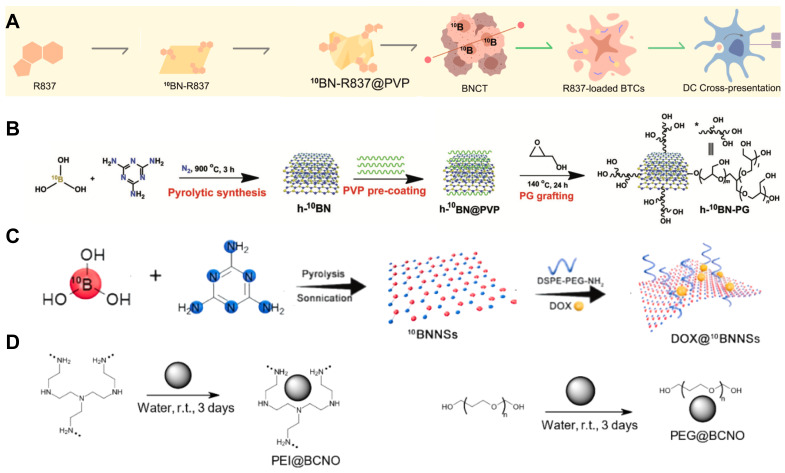
Designation of ^10^B containing BNCT nano-drugs with boron nitride. (A) ^10^BN-R837 @PVP is constructed with loading R837 on BN nanosheets for capturing neutrons and promoting antigen presentation. Reproduced with permission from 63. Copyright 2023, Elsevier. (B) Synthetic route of h-10BN-PG nanoparticles. Reproduced with permission from 64. Copyright 2023, Wiley-VCH GmbH. (C) Characterization of ultrathin 2D BNNSs and the loading capacity of DOX. Reproduced with permission from 65. Copyright 2021, Elsevier. (D) Schematic illustration of functionalization of BCNO nanoparticles with PEI and PEG. Reproduced with permission from 67. Copyright 2021, MDPI (Basel, Switzerland).

Boron nitride (BN) is a crystal composed of nitrogen and boron atoms. Its structure and properties are very similar to graphite, and can be used as high-temperature lubricants, heat shielding materials, etc. Boron nitride has several different crystal types, corresponding to different physical properties, so that it can be used in various application scenarios 58. One of the forms of existence of BN is boron nitride nanotubes (BNNTs), one of the structural analogues of carbon nanotubes (CNTs). It possesses excellent properties of CNTs such as thermal and chemical stability, electrical insulation properties. For BNNTs' peculiar shape, it can be used as a boron agent and also a nano drug carrier with high boron density which is suitable for BNCT. However, the BN nanoparticle is highly hydrophobic and not biocompatible, which leads to further surface modification. Nakamura *et al.* prepared a dispersed solution of BNNTs with the mPEG-DSPE protocol. Experiments proved that it could be significantly accumulated in B16 melanoma cells, and was a more effective boron carrier compared with BSH 59. In another study, a human epidermal growth factor receptor-2 (HER-2) targeting antibody was conjugated to a BNNT/β-1,3-glucan complex, which showed excellent deliverability and improved BNCT efficacy, confirmed its potential in clinical use (Figure 6A) 60. Similarly, Singh *et al.* also prepared a nanostructured boron nitride, which morphological examination showed that this boron nitride was in a transient state from two-dimensional hexagonal sheets to nanotubes. Different from ordinary boron nitride materials in the past, this nano-BN was synthesized at a relatively low temperature by modifying the synthesis techniques, which made it have high dispersion in water and be better absorbed by the body, so that it was more suitable for BNCT. Furthermore, the researchers validated its suitability for use in BNCT with different cell lines 61. Some other research strategies have made the safety and biocompatibility of boron carriers the primary concern. Li *et al.* designed a boron nitride nanoparticle (BNNP) that can degrade on demand (Figure 6B). A layer of phase-transitioned lysozyme (PTL) was coated on the outside of BNNPs, which could reduce its loss and prolong the blood circulation during drug transportation. After intravenous injection of BNNPs, vitamin C would be given subsequently to resolve the outer PTL, boosting the release of boron nanocrystals and its clearance in other major organs. These on-demand degradable BNNPs could be used in treating triple negative breast cancer, which is difficult to deal with using common treatment methods. With the high boron content and excellent biocompatibility, it could be considered a feasible solution for triple-negative breast cancer (Figure 7A) 62, 63.

In another study, it was reported ^10^B-enriched hexagonal-BNNPs grafted with poly(glycerol) could enhance the nanoparticles' stealth efficiency and accumulation in tumors, resulting in a high ^10^B concentration of 102.1 µg/g tumor cells and substantial eradication of CT26 tumor after one single injection and neutron radiation (Figure 7B) 64. Different forms of existence of boron nitride will also affect its function in BNCT. Li *et al.* fabricated boron nitride nanosheets (BNNS) as a nanocarrier to combine NCT and chemotherapy by loading doxorubicin on their surface (Figure 7C). This ultrathin nanosheet structure had great potential to add chemotherapy drugs inside and its boron-containing property could boost the drug release after giving low-energy neutron beam irradiation. Such combination treating method could enhance the therapeutic effect by inhibiting the self-repair behavior of cancer cells, which could be applied in cold tumors such as triple negative breast cancer. Both *in vitro* and *in vivo* experiments on this BNNS showed ideal antitumor effects, hinting that it could be seen as a promising nanostructure in BNCT 65. Boron carbon oxynitride (BCNO) is another family of boron nitride, which is a non-toxic and earth-friendly phosphor with application in high contrast cellular imaging and toxic metal sensors. Lan *et al.* prepared a polyethylene-glycol-coated boron carbon oxynitride nanoparticle (PEG@BCNO), it showed better therapeutic performance in animal models than that of ^10^BPA 66. Besides PEG@BCNO, Chiang *et al.* coated BCNO with polyethyleneimine (PEI), the PEI@BCNO exhibited higher boron content of 48μg B/g of tumor cell, while PEG@BCNO only reached 16μg ^10^B/g of tumor cell in *in-vitro* studies (Figure 7D) 67. However, these nanoparticles lack targeting ability towards tumor cells, so further surface modification with antibody or specific proteins might largely improve the tumor cell uptake of ^10^B, which leads to better clinical effects.

#### 2.2.3 Boron Carbide

**Figure 8:**
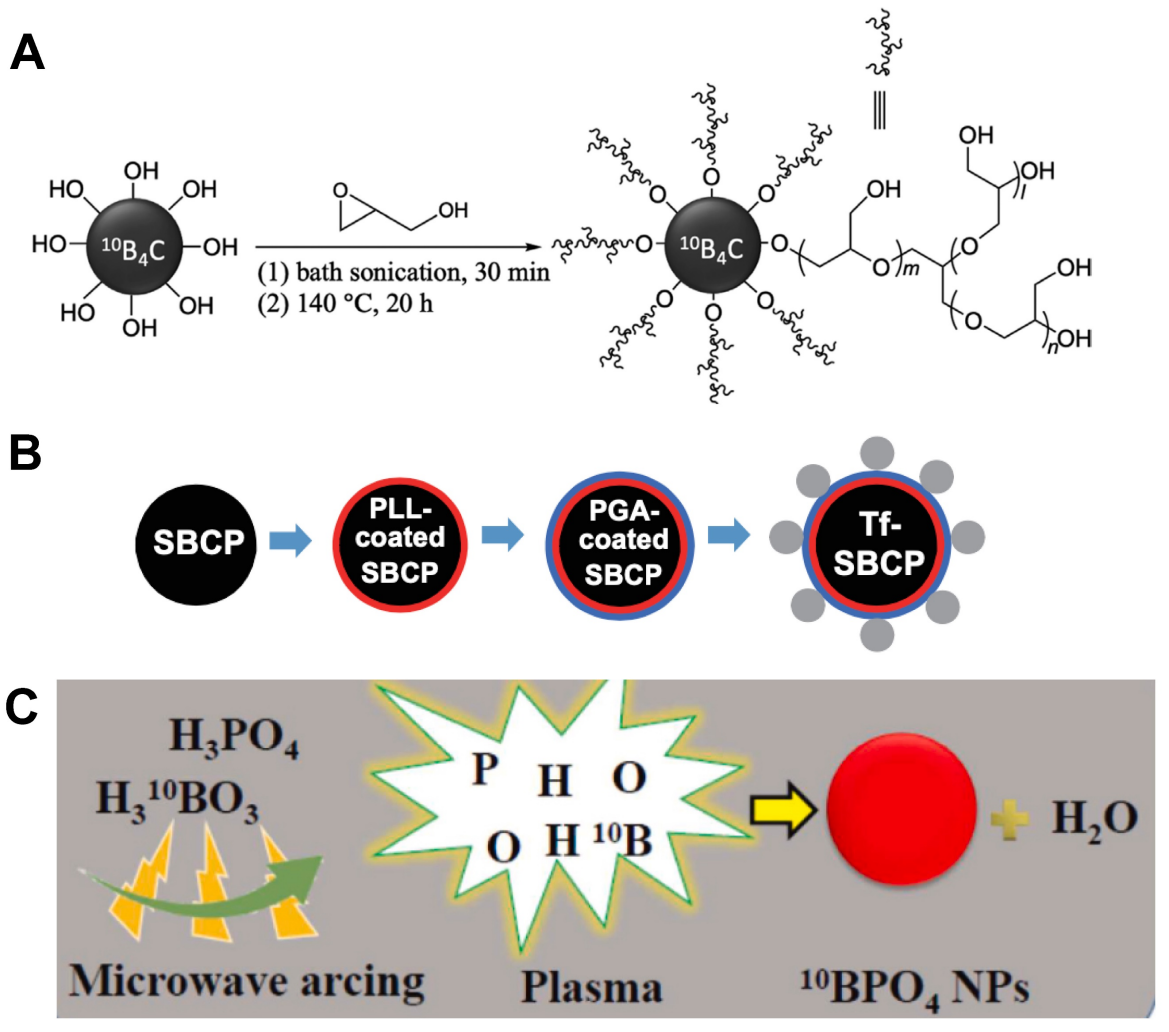
Designation of ^10^B containing BNCT nano-drugs with inorganic boron source. (A) Surface functionalization of ^10^B_4_C with hyperbranched PG through ring-opening polymerization. Reproduced with permission from 72. Copyright 2022, Wiley-VCH GmbH. (B) Schematic of spherical, submicrometer, Tf-modified B_4_C particles. Reproduced with permission from 74. Copyright 2018, Taylor & Francis. (C) Schematic presentation of synthesis of 10B-enriched ^10^BPO_4_ using a microwave arcing method. Reproduced with permission from 75. Copyright 2022, Elsevier.

Boron carbide is a covalent material which is characterized by outstanding mechanical properties, including a high melting point, low density, and high chemical resistance; when in the form of dense polycrystals, it possesses a high Young's modulus and extreme hardness 68. There are a large number of phases of boron carbide, such as B_6_C, B_2_C_2_, BC, B_4_C, B_13_C_2_ and so on. Most research on boron carbide focused on how to use simple methods to synthesize molecules or nanoparticles with high boron content 69, 70. Experiment showed that the obtained boron carbide nanoparticles presented good dispersion in water, and further surface modification would increase their targeting ability in clinical use 71. Some researchers tried to coat boron carbide nanoparticles with polymers to improve their biocompatibility and stability during the internal blood circulation, or graft antibodies on their surface to precisely target tumors and improve the uptake of ^10^B by tumor cells (Figure 8A) 72. *In vivo* studies proved that the boron carbide nanoparticles have the ability to suppress tumor growth, and the treatment was more effective when combined with other therapies (such as photothermal therapy) 73. Moreover, a kind of transferrin coated spherical boron carbide particles (Tf-SBCPs) was demonstrated by Tsuji and co-workers aiming at application in BNCT (Figure 8B). They invented a novel method to fabricate boron carbide particle of certain shape and size, which was helpful in medical application due to its homogeneous physical properties. Coating Tf outside the particles can offer them the ability of targeting cancer cells. From transmission electron micrographs, Tf-SBCP could be observed in a vesicle, indicating that the Tf-SBCP was internalised into the tumor cell via endocytosis. With the high boron content of boron carbide, Tf-SBCPs reached the prospecting boron concentration at a relatively low dose, and resulted in improved therapeutical effect 74.

#### 2.2.4 Other Inorganic Borides

Besides those inorganic compounds mentioned above, other common borides can also be applied in BNCT. One of the main problems in developing boron-containing nanoparticles is difficult to find a facile method which can synthesize size-controllable ^10^B-enriched nanoparticles. Kuthala *et al.* figured out a simple one-step synthesis of boron phosphate nanoparticle by using a microwave arching method, which resulted in an average particle size of about 100 nm (Figure 8C). The surface of this new nanoparticle was modified with antibodies that can target head and neck tumors, allowing them to be highly accumulated at the tumor site, in order to achieve a promising tumor-to-blood boron ratio of 4.27 75. There are also some creative researchers who transplanted techniques from other industries into the fabrication of boron-containing nanomedicine. Lin *et al.* synthesized fluorescent ^10^B embedded nanodiamonds using physical ion implantation technique that is mainly used in the semiconductor industry. Ion implantation is a well-developed technique that can place specific atoms into semiconductors in order to provide essential electrons and holes for electronic devices. Nanodiamonds belong to the category of nanoparticles which provide fantastic bio-compatibility and easily undergo surface modification with conventional chemical drugs that could kill tumor cells. Fluorescent ^10^B enabled real-time tracing of the delivery agents which is of vital importance to the process of BNCT, provided a promising boron delivery nanomedicine for not only BNCT but also variety biomedical applications 76.

## 3. Gadolinium-containing NCT Nanomedicine

Gadolinium (Gd) is one of the lanthanide elements, one of its isotopes, ^157^Gd, has a larger neutron capture cross-section of about sixty-six folds than that of ^10^B, enabling it to undergo a nuclear reaction after thermal neutron radiation, releasing high-energy gamma rays to kill tumor cells in distant tumor tissues and inhibit the growth of tumor at the same time. Meanwhile, Gd has the largest spin magnetic moment among all elements, so it is one of the most common clinical contrast agents in magnetic resonance imaging (MRI). The application of Gd to NCT (regarded as GdNCT) is expected to combine cancer treatment with MRI and further realize image-guided treatment, which has a promising application prospect. Previously, researchers had explored the therapeutic effect of ^10^B and ^157^Gd roughly by using BCo@CNPs and GdCo@CNPs, results showed that their cytotoxicity to HeLa cells are similar, and further *in vivo* study is needed for figuring out their potential in clinical use 77. Same as the discussion above, we will further analyze the application of gadolinium-containing nanomedicine in NCT by dividing gadolinium carrier agents into inorganic sources and organic sources.

### 3.1 Inorganic Gadolinium Sources

As one crucial metal element, Gd can form inorganic salts with almost all kinds of common acid ions. Among them, the oxides and chlorides of Gd are currently widely used in nanomaterials for NCT due to their plenty range of sources and simple preparation methods.

**Figure 9:**
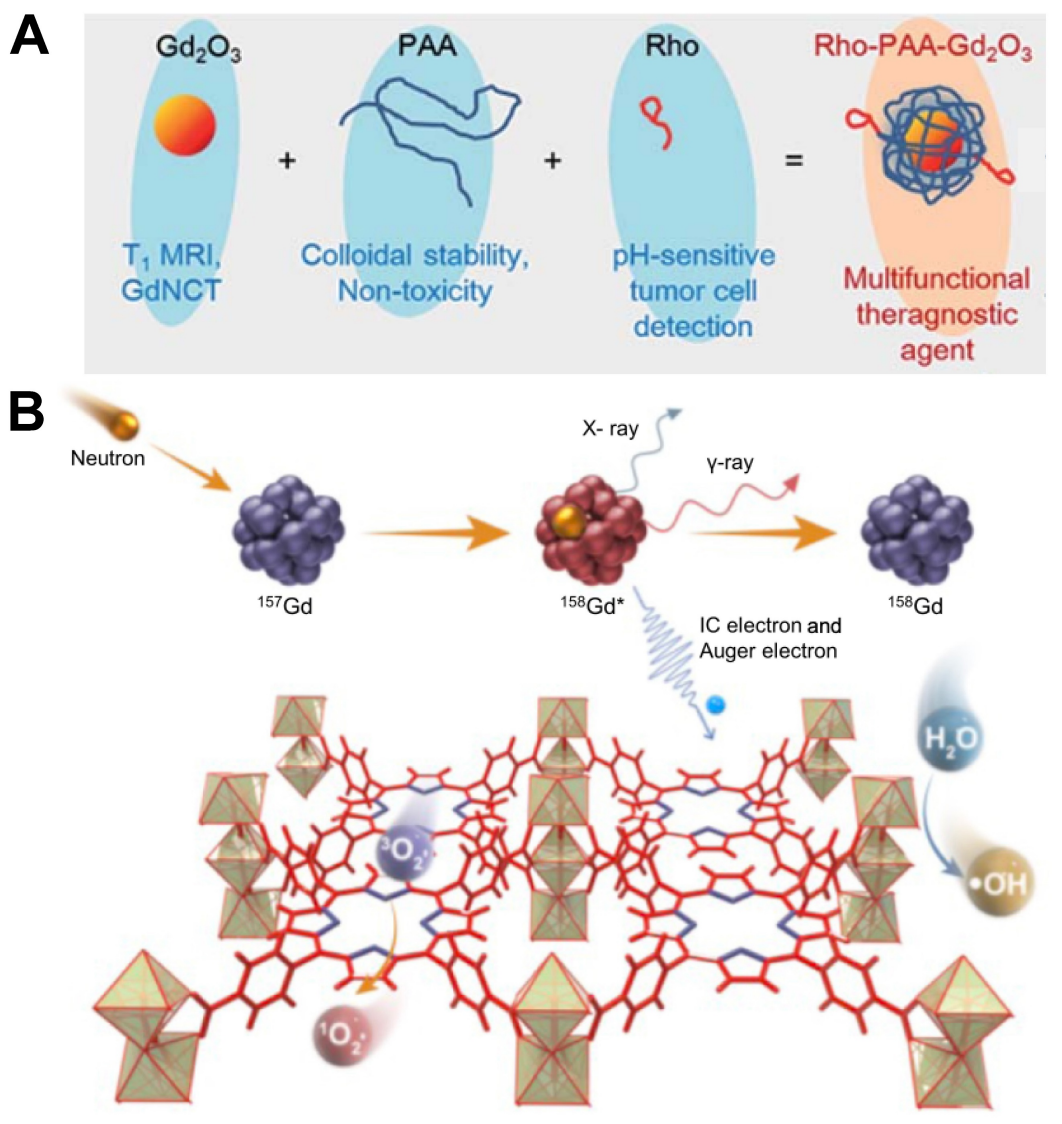
(A) Three components (i.e. ultrasmall Gd_2_O_3_ nanoparticle, PAA, and Rho) of the ultrasmall Gd_2_O_3_ nanoparticle colloid, the role of each component, and the surface coating structure. Reproduced with permission from 80. Copyright 2018, Royal Society of Chemistry. (B) Schematic illustration of the mechanism of GdNCT caused by Gd-TCPP. Reproduced with permission from 82. Copyright 2023, Chinese Chemical Society.

Gadolinium oxide, usually referred to as Gd_2_O_3_, is a kind of white solid with excellent stability and chemical inertness. It has a high melting point and can be used to prepare ceramic materials, optical glass and magnetic materials. The research group of Bridot has proved that the gadolinium oxide nanoparticles circulate in blood freely and cause no undesirable non-specific accumulation 78. A dose of at least 0.05mM ^157^Gd accompanied by 3 Gy neutron radiation could generate a cytotoxic effect to tumor cells but further* in vivo* studies are needed to prove its clinical validity 79. Ho *et al.* synthesized a monodisperse colloid of ultra-small gadolinium oxide nanoparticles (UGNPs) in 2018 (Figure 9A). Compared with macromolecular particles such as peptides, proteins, antibodies and so on, some small or ultra-small colloidal nanoparticles have higher number density, superior stability, and can more easily undergo surface-conjugation to enable them to possess additional functions. The ultra-small size of this nanoparticle with an average diameter around 1.5 nm facilitates its renal excretion and intravenous administration, and it can penetrate into the nucleus of cancer cells 80. As a contrast agent of MRI, the water proton relaxivity of UGNPs is higher, which means that the effect of enhancing contrast in imaging is greater. Based on those advantages, researchers combined UGNPs with polyacrylic acid (PAA) and rhodamine B, used PAA to wrap the colloidal particles to reduce its toxicity and improve the stability, then added a fluorescent dye rhodamine B to give it excellent fluorescence performance. This novel nanoparticle colloid shown excellent GdNCT therapeutic effect during *in vitro* and *in vivo* experiments, and was also effective in T_1_ MRI and pH-sensitive tumor cell detection, which could be regarded as one of the potential multifunctional tumor therapeutic nanomedicines. In addition to fluorescent dyes, tumor cell-targeting groups can also be modified on the ultra-small colloid particles to endow the nanomedicine with precise tumor delivery functions. Using the same ultra-small gadolinium oxide nanoparticle, the research team synthesized a new nanoparticle two years later by modifying it with a cancer-targeting ligand, arginylglycylaspartic acid (RGD) (shortly, RGD-PAA-UGNPs). Due to the property of tumor targeting, this nanomedicine could effectively accumulate in the tumor cells, increasing the Gd concentration at the tumor site, and it exhibited a significant therapeutic effect in the cancer model nude mice 81.

Gadolinium chloride can also be used as a Gd carrier in GdNCT. Liu *et al.* engineered a ^157^Gd-porphyrin framework structure using GdCl_3_⋅6H_2_O as a raw material (Figure 9B). Porphyrins are effective organic photosensitizers, which plays an important role in photodynamic therapy. Its main mechanism is to absorb laser energy to generate reactive oxygen species (ROS), so as to achieve the therapeutic purpose of killing tumor cells. Researchers combined ^157^Gd with porphyrin compounds, which was equivalent to combining GdNCT with photodynamic therapy. By generating ROS such as ⋅OH and ^1^O_2_ in tumor cells and cooperating with the high-energy rays produced by GdNCT, they achieved more effective cancer treatment outcomes in orthotopic and metastases animal models 82.

Inorganic Gd carriers have wide sources, low cost, and simple synthesis methods, which makes it possible to modify the physical properties to engineer nanomedicines for GdNCT. However, in the design and synthesis of nanomedicines, more attention should be paid to reducing the toxicity of Gd carriers and increasing their* in vivo* stability.

### 3.2 Organic Gadolinium Sources

Organic Gd carriers generally refer to chelates of Gd. Gadolinium chelates can be divided into two categories: linear chelates and macrocyclic chelates, in which macrocyclic gadolinium chelates are more stable than linear gadolinium chelates, and both can be used as contrast agents in MRI. We can also tell that almost all the contrast agents contain Gd have the potential to be used in NCT.

**Figure 10:**
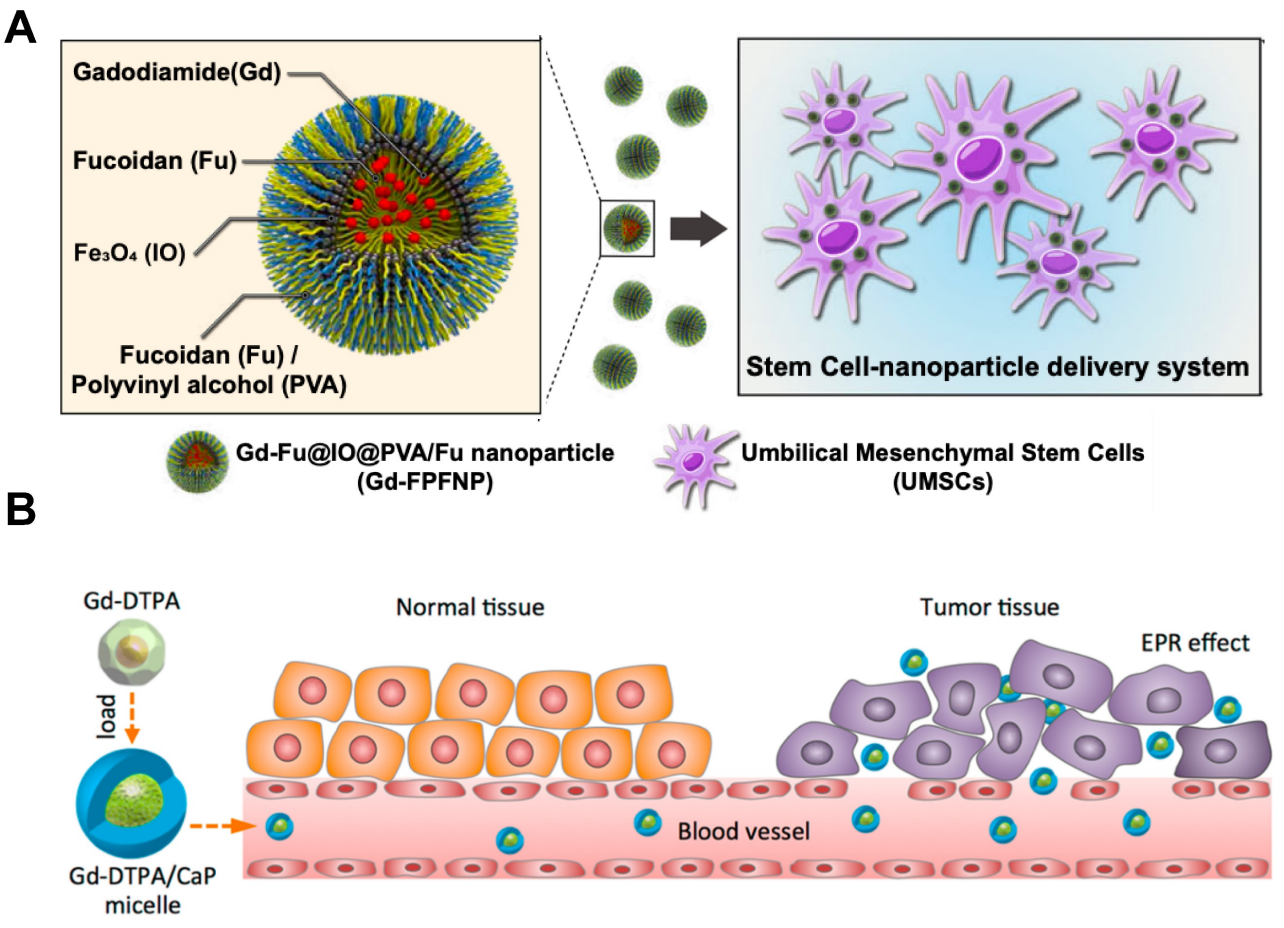
(A) Mechanism of action for stem cell-nanoparticle system (SNS) in gadolinium-neutron capture therapy (Gd-NCT). Reproduced with permission from 83. Copyright 2023, Springer Nature. (B) Scheme of Gd-DTPA/CaP hybrid micelles targeting tumors for gadolinium neutron capture therapy (GdNCT). Reproduced with permission from 84. Copyright 2015, American Chemical Society.

Lai *et al.* developed a stem cell-nanoparticle system (SNS) using gadodiamide as a starting material (Figure 10A). The gadodiamide molecule of nanoscale was wrapped in a dense shell composed of fucoidan and polyvinyl alcohol, iron oxide particles Fe_2_O_3_ were also incorporated in it, so that it could be transported to a specific site using magnetism. Finally, the nanoparticles were loaded into the umbilical cord mesenchymal stem cells (UMSCs), this kind of stem cells can penetrate the blood brain barrier and further target glioblastoma multiforme, so as to realize the precise delivery of tumor nanomedicine. The shell structure composed of fucoidan and polyvinyl alcohol could conceal gadodiamide, reducing its cytotoxicity towards UMSCs during drug delivery. In addition, fucoidan also has anti-inflammatory activity, and its application in the treatment of head and neck tumors could promote nerve repair after radiotherapy 83.

Most studies on NCT nanomedicines using Gd chelates as Gd sources have focused on their great potential to realize tumor MRI-guided internal radiation therapy. Mi *et al.* designed an adjuvant based on Gd-diethylenediaminepentaacetic acid (Gd-DTPA) combined with calcium phosphate micelles hybridized with PEG-polyanion block copolymers (Gd-DTPA/CaP) (Figure 10B). Herein, the outer part composed of calcium phosphate micelles could reduce the inherent toxicity of Gd-DTPA and improve its biocompatibility at the same time. After intravenous injection, the nanomedicine accumulated selectively in tumor cells and would not distribute systematically in normal tissue, distinguishing normal tissue from tumor so that improving the T/N ratio and T/B ratio. In addition, it also enhanced the selective contrast of MRI to tumor tissue, which could precisely locate the tumor and show great potential to implement radiotherapy safety 84. However, the authors attributed the selective uptake of the nanoparticle at the tumor site simply to the EPR effect without further explaining the mechanism of this tumor targeting phenomenon, which lacks scientific basis and persuasiveness. In the same year, Peters *et al.* also used Gd-DTPA to develop a new type of nanomedicine that could be available in GdNCT for the treatment of glioblastoma. Different from the above, this team used liposome-coated Gd-DTPA particles via the method of lipid/film-extrusion. The research team used rat glioblastoma cell F98 and human glioblastoma cell LN229 to carry out cell experiments in order to verify the feasibility of liposomes as nanocarriers. Results proved that this liposomal Gd-DTPA could effectively inhibit the survival and proliferation of these two tumor cells, and the therapeutic effect was related to the composition of liposomal and the concentration of Gd-containing nanomedicine 85.

More recently, other researchers also have used polymer nanocarriers to facilitate the accumulation in tumor cells and intracellular delivery of GdNCT nanomedicines under the guidance of MRI. Qin *et al.* used Gd-DOTA as the core part with polymer nanocarriers wrapping outside. These polymers were based on polyaspartic acid which were further modified with polyethylene glycol (PEG) chains of different molecular weights to control its stability and pharmacokinetics. *In vivo* experiments showed that PEG modification could promote the deposition of the nanomedicine in tumor tissues, and could effectively inhibit the growth of tumors after neutron irradiation. It could be regard as one of the nanomedicines with significant potential of application 86.

## 4. B-Gd Binary Nanomedicine

From the above summary we can tell, ^10^B and ^157^Gd are applicable isotopes that can be used in NCT. However, up to now, most research on NCT focused on one single isotope to explore its possibilities in tumor treatment. The hypothesis of nanoparticles containing both ^10^B and ^157^Gd that can take advantage of BNCT and GdNCT simultaneously is raised later on. Theoretically, GdBNCT enables MRI-guided internal radiation therapy, which can help identify the tumor size and location, making the diagnosis and treatment more precise and effective 87. Furthermore, due to the different mechanisms of BNCT and GdNCT, GdNCT has a longer ionization pathlength, which might be able to reach tumor at a further distance. Meanwhile, GdBNCT may provide a feasible solution for those locations where nanoparticles are difficult to deliver, such as hypoxic regions of tumors.

**Figure 11:**
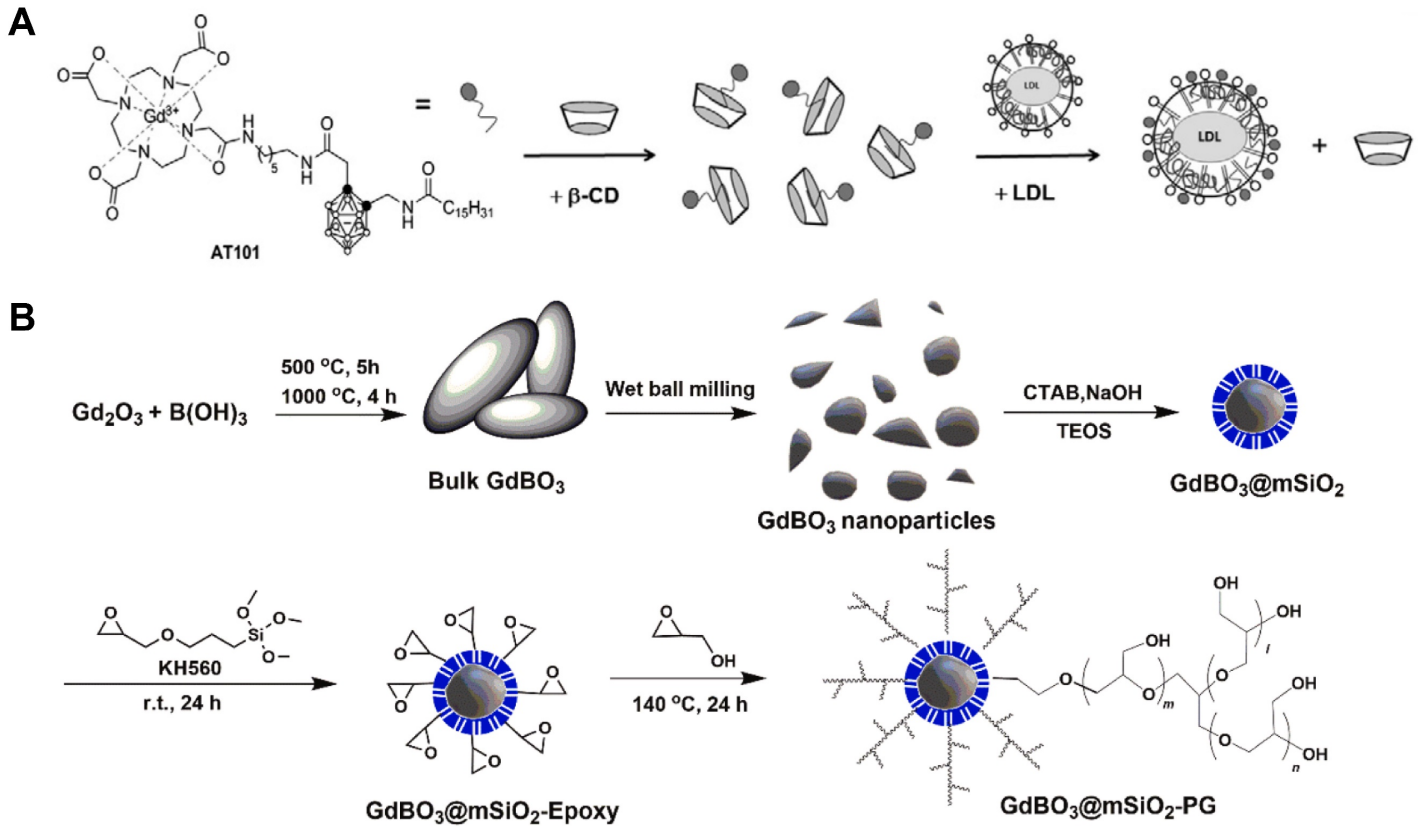
(A) Schematic depiction of the formation of AT101/β-CD and AT101/LDL adducts. Reproduced with permission from 88. Copyright 2015, Elsevier. (B) Preparation and surface modification of GdBO_3_ nanoparticles. Reproduced with permission from 90. Copyright 2022, Elsevier.

Alberti *et al.* designed a combination type of nanomedicine containing both boron and gadolinium using carborane and GdCl_3_. In order to master the boron concentration of tumor site, they attached carborane with Gd complexes, which could act as a MRI contrast agent to detect the boron concentration in real time with a noninvasive approach. Furthermore, considering the limited selectivity of boron delivery agents like BPA and BSH, they decorated the B/Gd agent with low-density lipoproteins (LDL), which could target the corresponding receptors on the surface of tumor cells (Figure 11A) 88. Apart from loading ^157^Gd onto boron nanoparticles, some compounds that originally contain these two isotopes can also be used in GdBNCT. Mikami *et al.* obtained gadolinium borate nanoparticles by hydrothermal method, they tested the cytotoxicity of the GdBO_3_ nanoparticle using human umbilical vein endothelial cells, which have been used for assessment of *in vitro* cytotoxicity abroad. No serious cytotoxicity effect has been shown at concentrations up to 300 µg/mL, suggesting great potential in clinical use, however, further *in vivo* studies are needed to confirm this assumption 89. Similarly, Zhang *et al.* prepared GdBO_3_ nanoparticles by wet ball milling, the nanoparticles were coated with mSiO_2_ and grafted by polyglycerol (Figure 11B). The final product GdBO_3_ @mSiO2-PG has excellent dispersibility and colloidal stability.* In vivo* retention and imaging capabilities have been demonstrated in this study, which suggests its potential application in GdBNCT, but therapeutic efficacy still needs to be explored 90.

**Figure 12:**
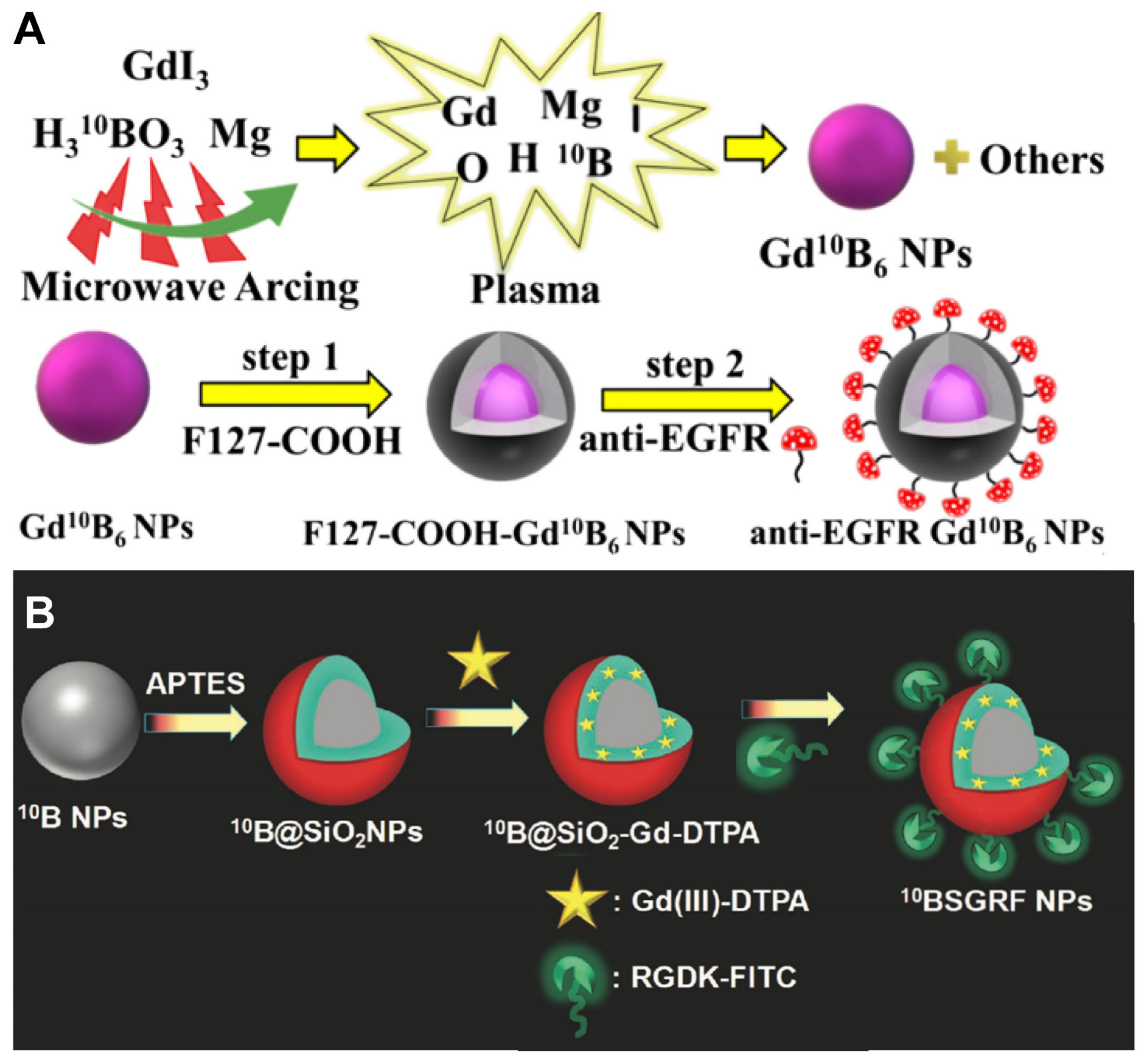
(A) Preparation of Gd^10^B_6_ NPs by a microwave arcing method and introducing tumor-targeting ability of Gd^10^B_6_ NPs by surface-grafting anti-EGFR moieties. Reproduced with permission from 91. Copyright 2023, American Chemical Society. (B) Schematic representation for the synthesis of the ^10^BSGRF NPs. Reproduced with permission from 92. Copyright 2017, Wiley-VCH GmbH.

In the literature, gadolinium hexaboride (GdB_6_) nanoparticles were synthesized through a unique modified microwave arcing method; the content of ^10^B in this Gd^10^B_6_ nanoparticle achieved 98.5%, which enabled it to deliver sufficient amount of ^10^B to tumor tissues and reach a fantastic T/B ^10^B ratio of 4.18 (Figure 12A). Furthermore, compared to the BPA-F treated group with a median overall survival of 38 days, the median overall survival of tumor-bearing mice was prolonged to 81 days after treated by GdBNCT using the Gd^10^B_6_ nanoparticles, showing an excellent prognosis 91. Similarly, Kuthala *et al.* demonstrated a ^10^B-enriched boron nanoparticle nanomedicine with 96% ^10^B content, which was surface-modified with fluorescein isothiocyanate (FITC)-labeled RGD-K peptide that can pass BBB and target glioblastoma (GBM) selectively (Figure 12B). The researchers incorporated Gd-DTPA complexes on the silica layer surfaces of this nanoparticle, so that it could also well-behave in the MR imaging and exploit the therapeutic effects of GdBNCT at the same time 92. One other related study showed several means of combination of ^10^B and ^157^Gd which can be used in Gd-guided BNCT, which pointed out that there are mainly two synthesis modes of this dual element agent, one was based on DOTA chelators and the other one was based on the structure of carborane cages 93. Different approaches to chemical synthesis could figure out the stability and toxicity of various types of these compounds. By trying diverse molecule structures, researches might hopefully explore the most effective way to obtain the greatest extent of the antitumor effect.

Studies on B-Gd binary nanomedicine are still at a preliminary stage with a small amounts and vague mechanisms. After neutron radiation, whether the principle of GdBNCT's function is consistent with that of BNCT and GdNCT alone remains to be further explored. Several researches mentioning this aspect also focused on the MRI ability of Gd complexes but not their therapeutic effects. Whether or not B-Gd binary nanomedicine can enhance the antitumor effect still lacks certain theory, so there is long way to go.

## 5. Discussion and Outlook

In this review, we systematically summarized the research progress of NCT nano-drugs. The various isotope types and sources were discussed first, followed by the attempts and endeavors of pertinent researchers in the past few years to combine nanomaterials with NCT. From this, we can get a glimpse of the development status and future development direction of neutron capture drugs, learn from many existing research results, and lay a solid foundation for more in-depth exploration in the future.

Nano-sized NCT medications have distinct physical and chemical features, a more efficient mode of administration, and more potential for developing treatments with excellent tumor targeting and strong biocompatibility when compared to typical NCT drugs incorporating boron. With the help of the high permeability and EPR effect of solid tumors, nanomedicines can also selectively accumulate in tumor cells. Nowadays, new drug synthesis methods and biomedical technologies are developing rapidly and emerging one after another, creating unique conditions for the innovation of NCT nanomedicines, especially nano-boron drugs. The design and development of nano-boron drugs can be combined with a range of nano-drug carriers, including inactivated viruses and engineered bacteria, new surface modification technologies, coating materials, etc. This gives broad ideas for the designation of nanomedicine with high tumor specificity and long-term retention in tumors.

In order to broaden the scope of this therapy's development and make it easier to implement multi-modal and multi-dimensional treatments—which can be thought of as combination therapy—the selection elements in NCT can be expanded vertically beyond boron. Taking GdNCT as an example, nanomedicine prepared from gadolinium can not only react with neutrons to kill tumor cells, but can also act as MRI tracers in the body to achieve MRI-guided NCT. Compared with traditional NCT, the assistance of imaging technology can enable medical staff to better grasp the concentration and enrichment location of drugs in the patients' body, making treatment more precise and effective. However, the current research on gadolinium-containing and boron-gadolinium binary nano-drugs is still in its infancy, and will be one of the important directions for the development of NCT nanomedicines in the future.

The development of NCT dates back almost a century. However, the advancement of this therapy has been sluggish during this time due to the constraints of its application scenarios and the technological obstacles to the production of pharmaceuticals containing boron. As a result, there is still a significant gap and opportunity for improvement in terms of clinical efficacy. Although the developmental advantages of nano-sized boron drugs are significant, there are also problems that need to be improved, such as long metabolism time and inability to be quickly eliminated from the body. Therefore, the synthesis of neutron capture nanomedicines with more ideal properties will be a research hotspot in the biomedical field in the future, and due to the particularity of the principle of NCT and the seriousness of the implications, it will also gradually enter the public's field of vision and provide important contributions to promote the development of the field of cancer treatment.

## Figures and Tables

**Figure 1:**
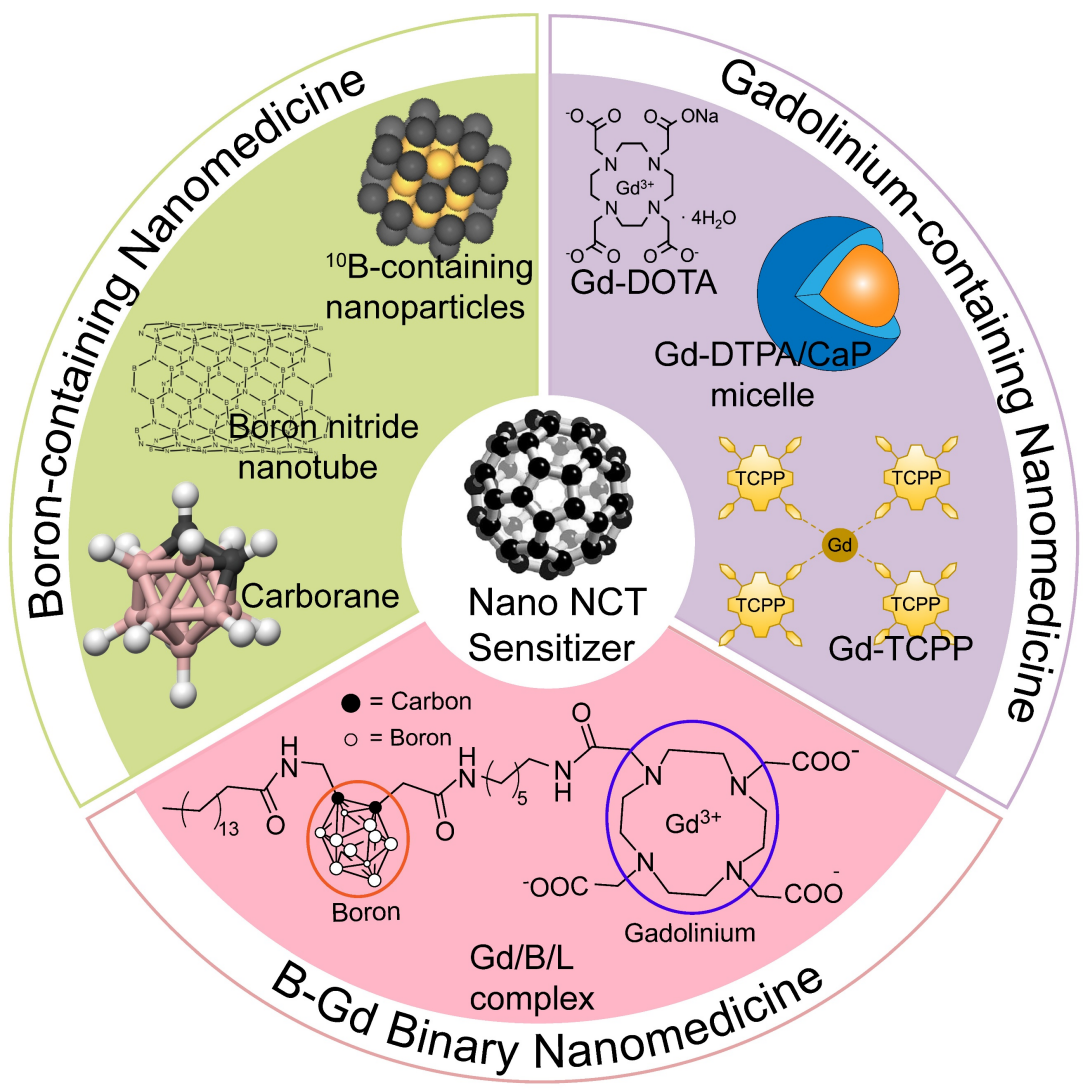
Schematic view of the classification and different types of nano-NCT sensitizers. Part of the figure is reproduced with permission from 59. Copyright 2014, Elsevier.

**Table 1:** Neutron capture cross sections of several common isotopes.

Isotope	Thermal neutron capture cross section (barn)
^1^H	0.33
^10^B	3.8 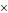 10^3^
^12^C	3.4 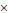 10^-3^
^14^N	1.8
^16^O	1.8 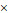 10^-4^
^155^Gd	6.1 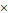 10^4^
^157^Gd	2.54 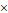 10^5^

## Abbreviations

ADP: adenosine diphosphate
APTMS: (3-aminopropyl)trimethoxysilane
ASGP-R: asialoglycoprotein receptor
BBB: blood-brain barrier
B-CHO: p-carborane-1,10-phenyl-dialdehyd
BCNO: boron carbon oxynitride
BN: Boron nitride
BNCT: boron neutron capture therapy
BNNP: boron nitride nanoparticle
BNNS: boron nitride nanosheets
BPA: 4-dihydroxyboryl-L-phenylalanine
BSH: sodium borocaptate
CDs: carbon dots
CNTs: carbon nanotubes
EGF: epidermal growth factor
EPR: enhanced permeability retention
F: fusion
FBY: fluoroboronotyrosine
FITC: fluorescein isothiocyanate
HER-2: human epidermal growth factor receptor-2
HN: hemagglutinin-neuraminidase
HVJ: hemagglutinating virus of Japan
GBM: gliblastoma
GdB_6_: gadolinium hexaboride
Gd-DTPA: Gd-diethylenediaminepentaacetic acid
GdNCT: gadolinium neutron capture therapy
LDL: low-density lipoproteins
MAC: K[*nido*-7-CH_3_(CH_2_)_15_-7,8-C_2_B_9_H_11_]
MEP: molecular electrostatic potential
MoAbs: monoclonal antibodies
MRI: magnetic resonance imaging
NCT: neutron capture therapy
PAA: polyacrylic acid
PAMAM: poly(amido)amine
PARP: poly ADP-ribose polymerase
PD: pharmacodynamics
PEG: polyethylene glycol
PET: positron emission tomography
PK: pharmacokinetics
PTL: phase-transitioned lysozyme
PVA: poly (vinyl alcohol)
ROS: reactive oxygen species
SNS: stem cell-nanoparticle system
SWCNTs: single-walled carbon nanotubes
TAC: Na_3_[1-(2′-B_10_H_9_)-2-NH_3_B_10_H_8_]
TAPB: 1,3,5-tris(4-aminophenyl)-benzene
Tf-SBCPs: transferrin coated spherical boron carbide particles
UGNPs: ultra-small gadolinium oxide nanoparticles
UMSCs: umbilical cord mesenchymal stem cells
